# Comparison of 3D quantitative osteoarthritis imaging biomarkers from paired CT and MR images: data from the IMI-APPROACH study

**DOI:** 10.1186/s12891-023-06187-2

**Published:** 2023-01-30

**Authors:** Alan Brett, Michael A. Bowes, Philip G. Conaghan

**Affiliations:** 1 Imorphics, Worthington House, Towers Business Park, Wilmslow Road, Manchester, M20 2HJ UK; 2 Leeds Institute of Rheumatic and Musculoskeletal Medicine, University of Leeds & NIHR Leeds Biomedical Research Centre, Leeds, UK

**Keywords:** Osteoarthritis, B-score, Biomarker, Machine learning, MRI, CT

## Abstract

**Introduction:**

MRI bone surface area and femoral bone shape (B-score) measures have been employed as quantitative endpoints in DMOAD clinical trials. Computerized Tomography (CT) imaging is more commonly used for 3D visualization of bony anatomy due to its high bone-soft tissue contrast. We aimed to compare CT and MRI assessments of 3D imaging biomarkers.

**Methods:**

We used baseline and 24-month image data from the IMI-APPROACH 2-year prospective cohort study. Femur and tibia were automatically segmented using active appearance models, a machine-learning method, to measure 3D bone shape, area and 3D joint space width (3DJSW). Linear regression was used to test for correlation between measures. Limits of agreement and bias were tested using Bland-Altman analysis.

**Results:**

CT-MR pairs of the same knee were available from 434 participants (78% female). B-scores from CT and MR were strongly correlated (CCC = 0.967) with minimal bias of 0.1 (SDD = 0.227). Area measures were also correlated but showed a consistent bias (MR smaller). 3DJSW showed different biases (MR larger) in both lateral and medial compartments.

**Discussion:**

The strong correlation and small B-score bias suggests that B-score may be measured reliably using either modality. It is likely that the bone surface identified using MR and CT will be at slightly different positions within the bone/cartilage boundary. The negative bone area bias suggests the MR bone boundary is inside the CT boundary producing smaller areas for MR, consistent with the positive 3DJSW bias. The lateral-medial 3DJSW difference is possibly due to a difference in knee pose during acquisition (extended for CT, flexed for MR).

**Trial registration:**

NCT03883568

**Supplementary Information:**

The online version contains supplementary material available at 10.1186/s12891-023-06187-2.

## Background

Osteoarthritis (OA) of the knee is a serious disease resulting in pain, loss of function and reduced quality of life; it is a leading cause of disability among older adults [1–3]. The pathophysiology of OA involves multiple tissues, with deterioration of both cartilage and bone considered integral to the OA process [4, 5]. The primary structural assessment of OA is often based on radiographs in which pathologic changes such as osteophytes are taken as the first signs of disease. Other bony changes (sclerosis and deformation of the bone contour) may be visualized at later stages of the disease and are included with osteophytes in the semiquantitative Kellgren- Lawrence grade (KLG) [6], the most commonly-used OA radiographic scoring system. However, radiography is relatively insensitive in detecting earlier changes and is imprecise due to its dependence on both acquisition method and reader [7].

The use of Magnetic Resonance Imaging (MRI) in the study of OA has not only enabled a more thorough understanding of the anatomy and pathology of the OA joint in three-dimensions, it also provides a detailed visualization of multiple tissues with excellent soft-tissue contrast. Several MRI studies using semiquantitative scoring systems have demonstrated pathology associated with OA in knees with normal radiographs. The presence of MRI-detected osteophytes has been shown to occur in 74% of knees with normal radiographs and the prevalence of “any abnormality” has been found to be as high as 89% [8].

Quantitative assessment of MR images can provide direct measurement of cartilage and bone [9], and a number of quantitative 3D MR imaging biomarkers for the assessment of knee OA have been developed and validated using large MR image datasets [10]. MRI bone morphology changes in subchondral surface area [11, 12] and parameterized shape descriptions [13, 14] have been employed as endpoints in DMOAD clinical trials [15, 16].

Statistical shape modelling (SSM), a type of supervised machine-learning, allows for the parametrization of complex 3D anatomical shapes such as the knee [17]. The method can be employed both to describe and compare knee shape; and to automatically search for and segment a knee shape in an image, enabling the analysis of very large image datasets [18]. Measurement of bone area and shape changes from MR images using SSMs has been shown to predict radiographic onset of OA [19], is associated with radiographic structural progression [20] and discriminates knees with OA from non-OA [21].

Recently, Bowes et al. [13] introduced a method for the reduction of the 3D shape of femur to a single metric value termed the “B-score”. This statistical z-score metric compares the shape of non-OA and OA knees on a continuous linear scale with an origin at 0 (KLG 0 middle-aged knee) and a unit scale based on the standard deviation of these KLG 0 knees. The approximate observable B-score range is from −3 to +7 with advancing OA in the positive direction. The B-score has been shown to be an objective, automated assessment of OA status with clinical risk defined for current and future pain, functional limitation and Total Knee Replacement (TKR) [13].

Radiographic Joint space width (JSW) measured between the femoral and tibial margins on a weight-bearing x-ray is widely accepted as an indicator of knee joint health. Because the radiograph is a 2D projection of a 3D structure, measurements are highly dependent on the acquisition conditions, such as the position of the knee, knee flexion angle, and the alignment of the X-ray beam with the tibial plateau. Even with carefully followed protocols using customized knee positioning devices and optimal acquisition conditions, conventional radiographs can remain insensitive, inaccurate, and have poor concurrent validity for knee OA features [22]. In contrast, the measurement of 3D JSW using tomographic imaging is unencumbered by the problem of overlapping anatomy and reproducible anatomical alignment. 3D JSW therefore represents a relatively novel OA imaging biomarker that is readily available from MR images that are acquired for quantitative assessment of cartilage and bone in DMOAD trials. Measurement of 3D JSW has been previously demonstrated using weight-bearing CT images [23, 24] though this has not been performed using supine MR and CT images.

While the widespread use of MRI for clinical trials and research in OA has led to bone imaging biomarkers being developed for this modality, MR is more usually employed for soft-tissue and trabecular bone evaluation since MR imaging pulse sequences usually depict cortical bone as a signal void. Computerized Tomography (CT) imaging is more commonly used for 3D visualization of bony anatomy due to its high bone-soft tissue contrast [25]. In addition, CT images are not subject to the geometric distortion that can be caused in MR images due to magnetic field inhomogeneities [26], a potential problem that is addressed in DMOAD clinical trials by the careful and consistent positioning of the knee near the isocenter of the magnet, where the field is most homogeneous.

To test the robustness to the choice of imaging modality of the B-score as a measure of femoral cortical bone shape in DMOAD clinical trials, we compared the results of automated analysis of CT-MR image pairs acquired at baseline and 24-month timepoints. We also compared a similar bone shape z-score of the tibia, and measures of bone area from femur and tibia, and medial and lateral tibiofemoral 3DJSW.

## Methods

### Subject image data

We used image data from the IMI “Applied Public-Private Research enabling OsteoArthritis Clinical Headway” (APPROACH) study. APPROACH is an exploratory, European, 5-centre, 2-year prospective longitudinal cohort study. It includes clinical, imaging, biomechanical and biochemical parameters, in a cohort of 297 participants (age; 66.5 ± 7.1, female; 230 (77%), BMI; 28.1 ± 5.3) recruited primarily from prior European OA cohorts using machine learning models based on retrospective patient data to exhibit a high likelihood of radiographic JSW loss and/or knee pain over the course of the study [27]. The study has been approved by the Ethical Committees of the participating countries and has been registered in Clinicaltrials.gov (NCT03883568).

Low-dose CT images (120kVp, 220 mAs, 0.625 mm slices, 0.3 mm in-plane resolution, medium kernel) were acquired at 5 sites for 271 participants at baseline and 215 participants at 24-months. Both knees were positioned to be as straight as possible, with the feet on a wedge to produce around 15^o^ of internal rotation at the hip. These CT images were matched with sagittal 3D WATS, 3D FFE, or 3D FLASH MR images (1.5 mm slice thickness; 0.31 mm in-plane resolution; TR 17 ms, TE 7 ms, FA 12–15° with fat-saturation or water excitation) of the single index (left or right) knee for each subject acquired using 1.5 T or 3 T MR systems. MR acquisition protocol was to rotate the leg so that the toes and patella faced upward, elevate knee by putting cushions below it so that the center of the knee is place in the center of the coil and avoid scanning the knee in a fully extended or hyperextended position. CT and MR images were acquired within a mean of 12 (median: 0, max: 162) days of each other. Additional test-retest sagittal 3D WATS, 3D FFE, or 3D FLASH MR images with repositioning were available from 37 participants at baseline (8), 6-month (16) and 24-month (13) timepoints. KLG was read centrally from standard weight-bearing x-rays taken at the screening visit [27].

### Statistical shape modelling & image search

Femur and tibia bones were automatically segmented from MR images using active appearance models (AAMs), a type of SSM trained to search images, provided by Imorphics (Manchester, UK). AAMs are proven technology that can segment knee bone surfaces with sub-millimeter accuracy as has been described previously [21, 28]. AAMs were constructed using a training set, from 3D high-resolution water-excitation MR images, selected to provide examples of all stages of OA [29].

A similar AAM was constructed for the automated search of CT images with a second-stage refinement using convolutional neural network machine-learning [30]. The AAM was trained on manual segmentations of 122 CT images displaying varying KLG grades, and the CNN was trained on a separate set of 3500 pre-operative CT images from a database of CT scans acquired robotic total knee replacement surgery [31]. Segmentation model accuracy was validated using another independent set of 1097 pre-operative images and expert manual segmentations. Measurement accuracy was assessed using the mean absolute point-to-surface distance between model and manual segmentations. Mean segmentation errors were 0.12 mm (standard deviation (SD)) 0.04 mm (mm) for femora and 0.13 (0.08) mm for tibiae.

### Bone shape

We constructed an ‘OA vector’, defined as the line passing through the mean shape of a population with OA (OA Group, defined as all knees with KLG ≥2 at all four time points of 0, 1, 2 and 4 years) and a population without OA (Non-OA Group, defined as those with KLG of 0 at each of the same time points). For the femur, distances along the OA vector have previously been termed ‘B-score’ [13], with the origin (B-score 0) defined as the mean shape of the Non-OA Group for each sex. The B-score has the form of a statistical z-score so that 1 unit is defined as 1 SD of the Non-OA Group along the OA vector (positive values towards the OA Group) [13]. A similar bone shape z-score was constructed on an OA-vector for the tibia for each sex.

We used the same femur and tibia OA-vectors constructed using MR images to produce both the MR and CT image bone shape results. For the femur, this was the previously described shape vector used to produce the B-score. The OA-vector for the tibia has also been previously described [12]. When analyzing a CT image, the resulting bone shape surface from the CT AAM search was searched for a best fit using a version of the MR AAM trained to fit to a predefined surface shape. This process produces an MR AAM shape instance with the correct MR model landmark points but with the shape of the CT image search result. This MR model shape instance can then be projected onto the previously defined MR OA-vectors for femur or tibia. To determine the accuracy of this step, we measured the mean of absolute point-to-surface distances between the CT AAM search result and the derived MR AAM shape instance across all available baseline and 24-month CT-MR image pairs.

### Bone area

Anatomical regions of total area of subchondral bone (tAB) [32] representing medial and lateral femur (MF, LF), medial and lateral femoral trochlea (TrFMed, TrFLat) and medal and lateral tibia (MT, LT) were outlined on the mean AAM bone shape as previously described [21]. The boundaries of the MF.tAB and LF.tAB regions and trochlea grove were defined as a line on the bone corresponding to the anterior edge of the medial or lateral meniscus in the mean shape. During auto-segmentation with AAMs, these regions are automatically propagated to each bone surface, allowing for the measurement of anatomically corresponded tAB regions on the knee bone surfaces from each subject. The six regions originally defined on the surface mesh of the mean MR AAM shape were then projected to the surface mesh of mean CT AAM shape by registering the two surface meshes using Iterative Closest Point (ICP) algorithm [33].

### 3D joint space width

**Fig. 1:**
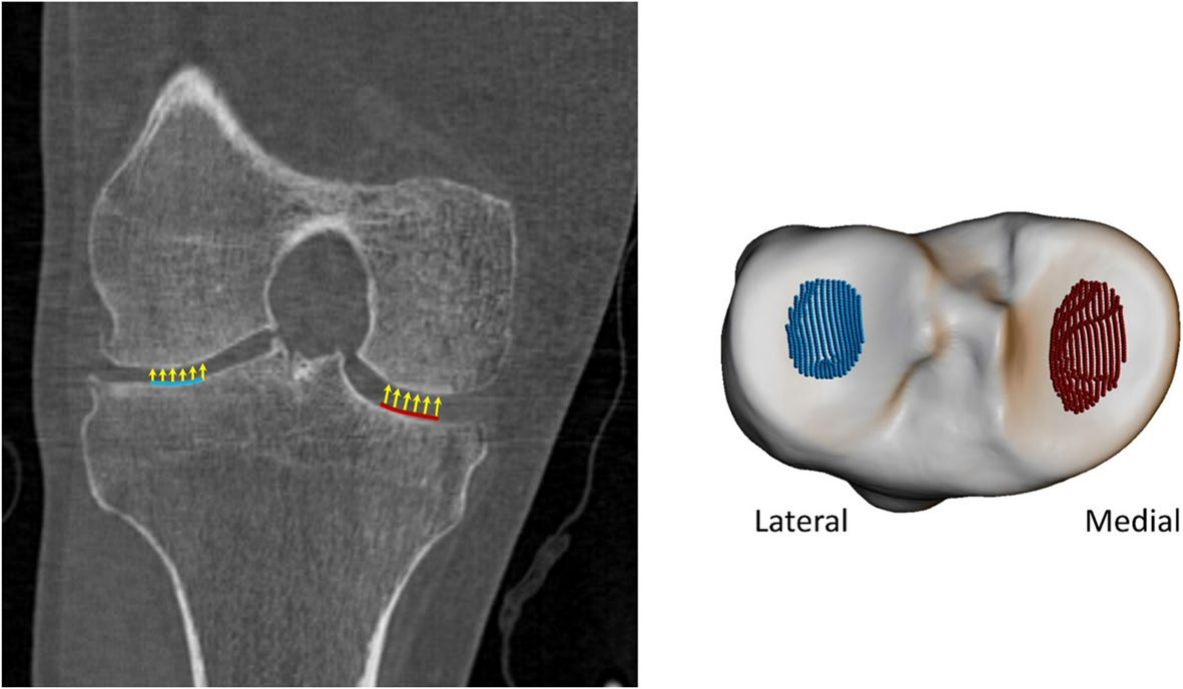
Three-dimensional measurement of central medial and lateral mean joint space width (3DJSW). The central medial region is shown in red, and the lateral region is shown in blue (right figure). The coronal view of a typical knee is shown in the left figure, which shows where these two regions are located, and how measurements are taken, normal to the 3D tibial surface

3D JSW measurements (3DJSW) were produced for all the landmark points enclosed by central regions defined on the medial and lateral tibial plateaus (Fig. 1). Each region consists of approximately 2000 landmarks and were defined on the surface mesh of the mean shape of the MR AAM as the cLT and cMT regions previously described anatomically for manual cartilage measurement [34]. These regions were then projected to the surface mesh of mean CT AAM shape by registering the two surface meshes using Iterative Closest Point (ICP) algorithm [33]. During auto-segmentation with AAMs, these regions are automatically propagated to each bone surface, allowing for the measurement of 3DJSW at anatomically corresponding positions in each subject. Measurements at each point were made between the segmented tibial and femoral mesh surfaces along a normal to the tibial surface. An average 3DJSW was then computed from the measurements at all points in the medial or lateral region.

### Statistical analysis

We used linear regression and Lin’s Concordance Correlation Coefficient (CCC) [35] to test for correlation between the MR and CT measures of bone shape, area and 3DJSW. Limits of agreement and systematic bias was tested using Bland-Altman analysis of these measures. Analysis was performed on both the full dataset incorporating all available KLG scores and on a limited subset incorporating only KLG 2 & 3 scores, since these would typically be chosen for inclusion in a DMOAD trial. Smallest detectable difference (SDD) of the two bone shape measures was computed from test-retest image pairs using Bland-Altman analysis. Images acquired from the same subject but at baseline or 24-month timepoints were treated as independent.

## Results

Baseline and 24-month CT-MR image pairs of the same knee laterality were available from 231 and 203 participants respectively, resulting in 434 CT-MR image pairs for analysis. In the analysis set, there were 338 female knees (78%). Kellgren-Lawrence Grading for the analyzed (index) knee in these participants was KLG 0 (19%); KLG 1 (31%); KLG 2 (30%); KLG 3 (16%); KLG 4 (3%) with 5 knees ungraded (1%), providing coverage across the OA spectrum. The limited subset incorporating KLG 2 & 3 knees comprised 197 CT-MR pairs with KLG 2 (65%) and KLG 3 (35%).

Mean time between CT and MR image acquisition was 10 (median: 0 days; 95th percentile: 116) days. At least half of the MR-CT scans were acquired on the same day, and 95% were acquired within 4 months of each other, a period of time which is unlikely to result in measurable anatomical change. In general, where there was a time between acquisition of modalities, the CT acquisition came later. SDD of the B-score measure from test-retest comparison was 0.227 which is comparable to the SDD of 0.251 measured previously [13]. For the tibia z-score, SDD was 0.373. The mean (SD) of absolute point-to-surface fitting errors between the CT AAM search result and the derived MR AAM shape instance across all available baseline and 24-month CT-MR image pairs was 0.098 (0.022) mm.

**Fig. 2:**
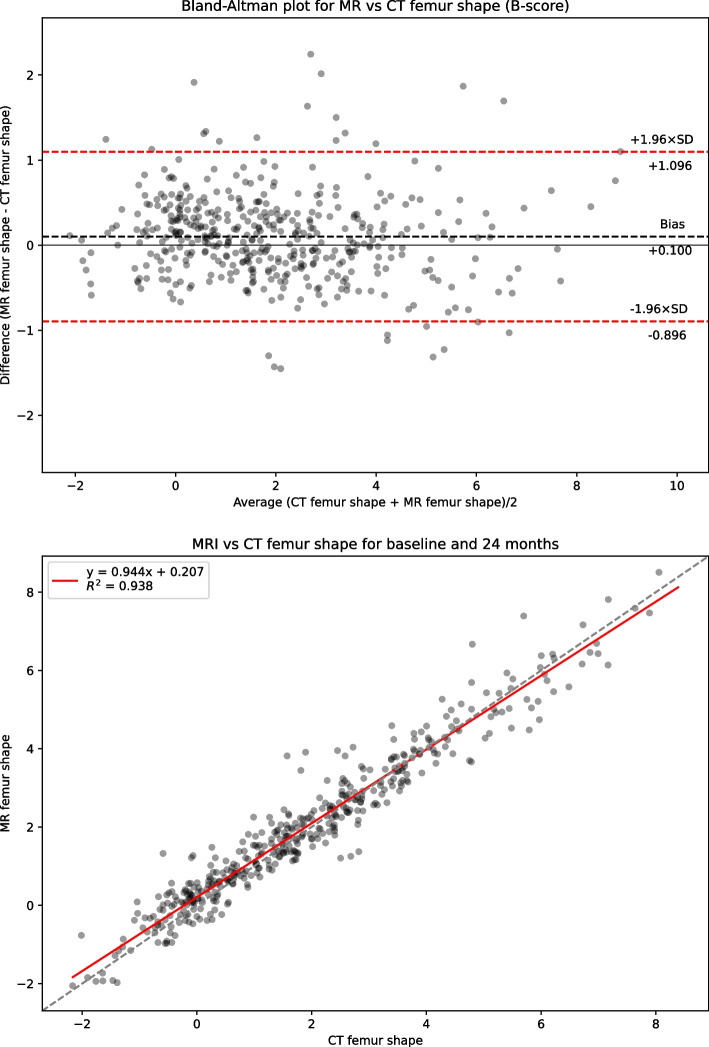
CT vs MRI B-score for the full dataset (KLG 0–4). Top: Bland-Altman (MR minus CT) plot; Bottom: linear regression

**Fig. 3:**
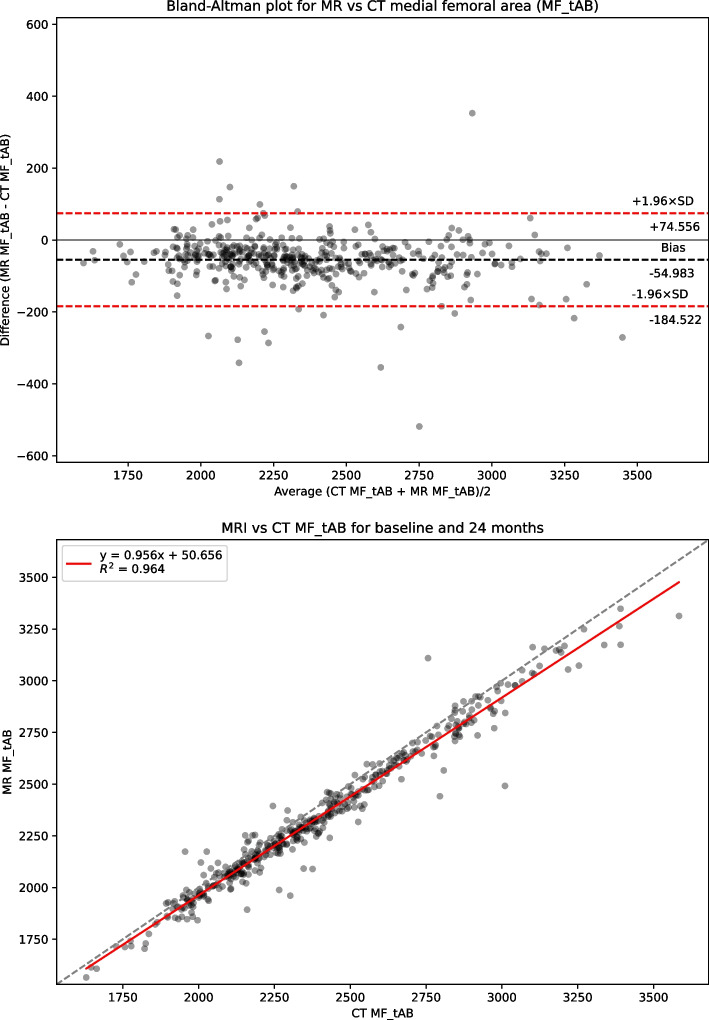
CT vs MRI bone area MF.tAB for the full dataset (KLG 0–4). Top: Bland-Altman (MR minus CT) plot; Bottom: linear regression

**Fig. 4:**
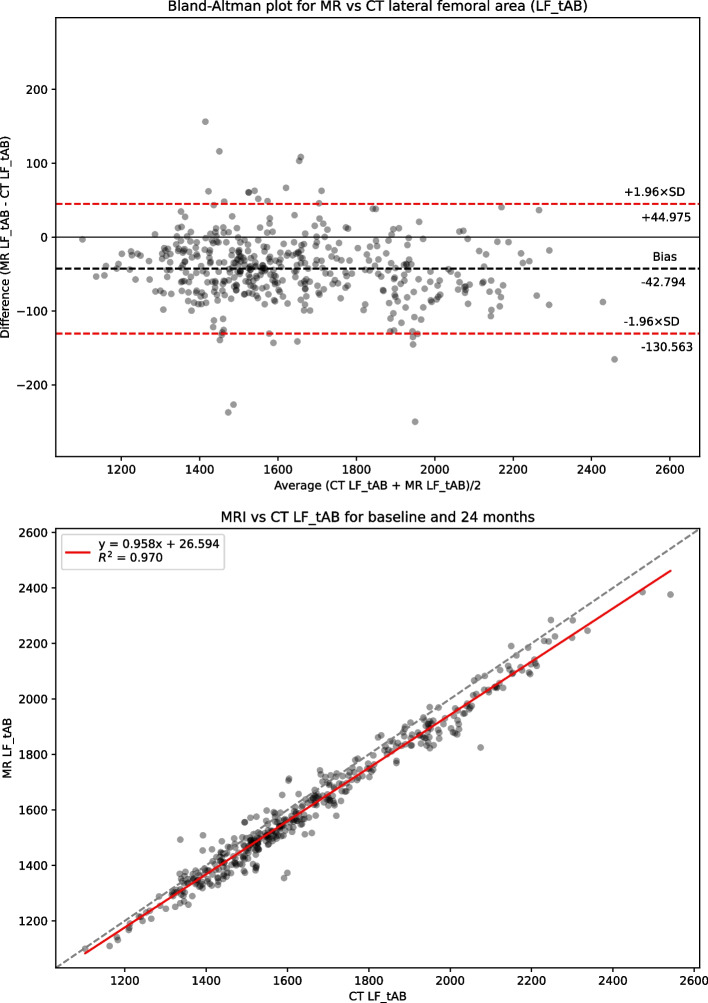
CT vs MRI bone area LF.tAB for the full dataset (KLG 0–4). Top: Bland-Altman (MR minus CT) plot; Bottom: linear regression

**Fig. 5:**
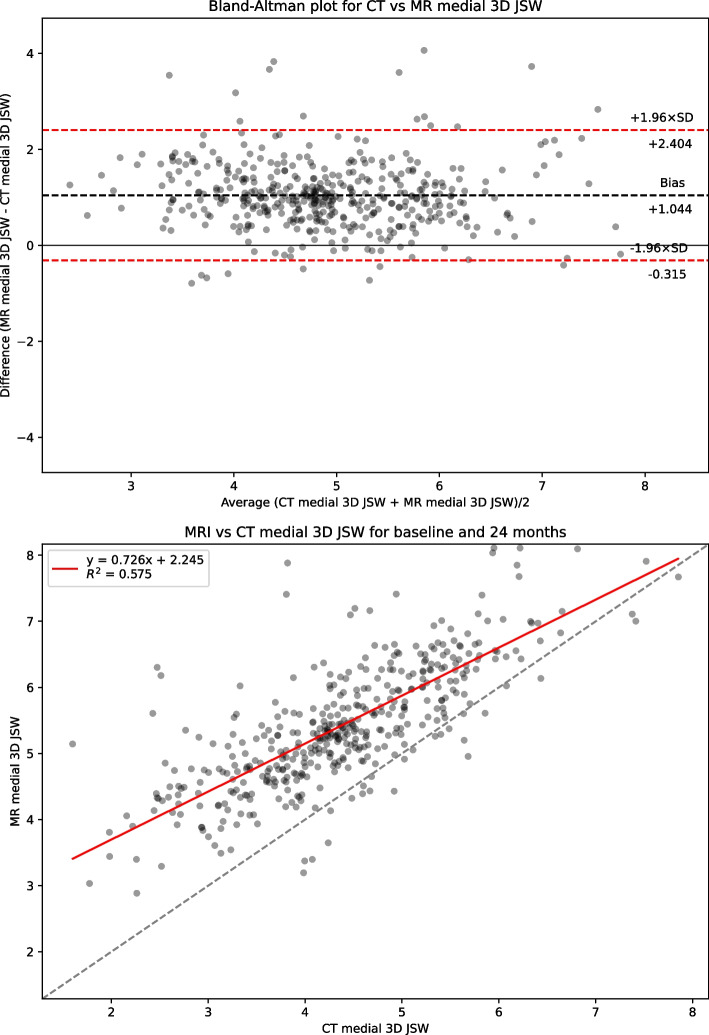
CT vs MRI medial 3DJSW for the full dataset (KLG 0–4). Top: Bland-Altman (MR minus CT) plot; Bottom: linear regression

**Fig. 6:**
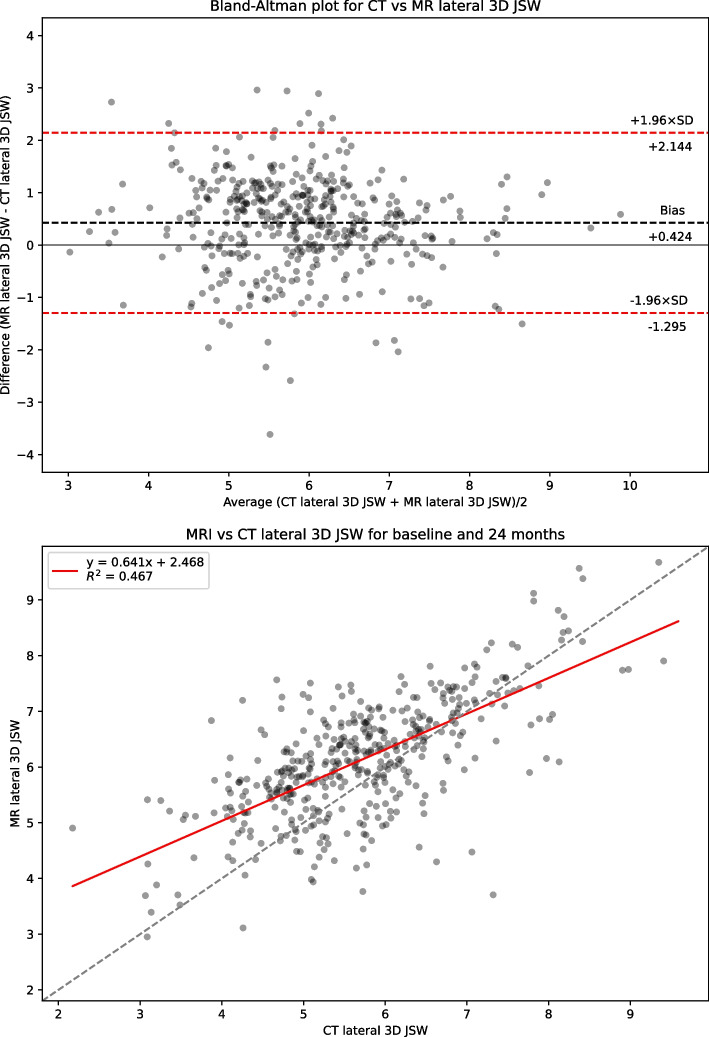
CT vs MRI lateral 3DJSW. Top: Bland-Altman (MR minus CT) plot; Bottom: linear regression

Table 1 presents summary statistics for shape, area and 3DJSW measures: Bland-Altman bias with 95% confidence intervals, Bland-Altman limits of agreement, linear regression coefficient of determination R^2^, and Concordance Correlation Coefficient (CCC) for the full dataset. Summary statistics for the limited KLG 2 &3 dataset is presented in supplemental Table S1. Figure 2 shows linear regression and Bland-Altman analysis for B-score. Figures 3 and 4 show linear regression and Bland-Altman analysis for bone areas MF.tAB and LF.tAB. Figures 5 and 6 show linear regression and Bland-Altman analysis for medial and lateral 3DJSW. Results for trochlea bone area, tibia shape, and tibia bone area are provided in supplemental Figs. S1-S5.

**Table 1:** Bland-Altman bias (MR minus CT), limits of agreement and linear regression statistics for the comparison of various MR and CT derived measures for the full dataset (KLG 0–4)

Measure	Bland-Altman bias [95% CI]	Bland-Altman limits of agreement		R^2^	CCC
		Lower [95% CI]	Upper [95% CI]		
**Femur shape** **B-score**	0.100 [0.052, 0.140]	−0.896 [−0.979, −0.813]	1.096 [1.013, 1179]	0.938	0.967
**Tibia shape** **z-score**	−0.424 [−0.475, −0.374]	−1.471 [−1.558, −1.384]	0.622 [0.535, 0.709]	0.910	0.924
**MF.tAB** **(mm**^**2**^**)**	−54.983 [−61.218, −48.748]	−184.522 [−195.322, −173.722]	74.556 [63.756, 85.356]	0.964	0.969
**LF.tAB** **(mm**^**2**^**)**	−42.792 [−47.019, −38.569]	−130.563 [−137.880, −123.245]	44.975 [37.657, 52.292]	0.970	0.971
**MT.tAB** **(mm**^**2**^**)**	−5.858 [−9.235, −2.482]	−75.997 [−81.84, −70.149]	64.380 [58.432, 70.128]	0.962	0.978
**LT.tAB** **(mm**^**2**^**)**	−21.329 [−23.896, −18.762]	−74.659 [−79.106, −70.213]	32.001 [27.555, 36.447]	0.965	0.968
**TrFMed.tAB** **(mm**^**2**^**)**	−28.346 [−30.714, −25.978]	−77.544 [−81.646, −73.443]	20.853 [+16.751, +24.955]	0.932	0.918
**TrFLat.tAB** **(mm**^**2**^**)**	−39.387 [−43.002, −35.773]	−114.477 [−120.738, −108.217]	35.703 [29.442, 41.963]	0.947	0.945
**Medial 3DJSW** **(mm)**	1.044 [0.979, 1.110]	−0.315 [−0.428, −0.202]	2.404 [2.290, 2.517]	0.575	0.635
**Lateral 3DJSW** **(mm)**	0.424 [0.342, 0.507]	−1.295 [−1.438, −1.151]	2.144 [2.000, 2.287]	0.467	0.489

B-scores measured using CT or MR images were strongly correlated (CCC = 0.967) and showed good agreement with a small MR positive bias of 0.100 [95% CI: 0.052, 0.14]. Limits of agreement (LOA) for B-score were − 0.896 and + 1.096 which is significantly higher range than the SDD of +/− 0.227. Although the tibia z-score measures were also highly correlated (CCC = 0.924), the agreement was less good with a negative bias of −0.424 [−0.475, −0.374]. All bone area measures were also highly correlated between CT and MR with a consistent negative bias (MR measures smaller). The 3DJSW measures were less well correlated and both larger were for MR than for CT but had a different bias in the lateral of 1.044 [0.979, 1.110] mm and compared to a bias of 0.424 [0.342, 0.507] mm in the medial side. Bland-Altman plots for all measures showed little or no apparent correlation and so did not indicate any association between measurement values and bias.

## Discussion

B-score measures from MR or CT images were strongly correlated and showed good agreement with a relatively small bias of 0.1, suggesting that bone shape may be reliably measured using either modality. This bias could be applied to CT B-score measures as a small positive correction. However, the bias is considerably smaller than the SDD of 0.227 measured from MR images, and the linear regression model crosses the unity line indicating that this bias is positive (MR larger) for the lower B-scores and negative (CT larger) for the higher B-scores. Therefore, this small correction is probably not significant in most practical applications when measuring B-scores using CT images. In the limited subset analysis of KLG 2&3 knees, the B-score bias was reduced to 0.049, suggesting that for clinical trials incorporating such a a cohort, the potential difference obtained for the measure from the two modalities is probably not significant. In fact, this represents about 12 months of expected B-score change in a non-OA cohort [16].

The Bland-Altman limits of agreement between CT and MR measurements are relatively large compared to the SDD derived from the MR test-retest image data. This increased variability when comparing B-scores measures from CT and MR is unlikely to be due to the slightly higher sample variance in the CT measurement (4.15 compared to 3.95 for MR measurement). An observable difference in variance would produce correlation structure in the Bland-Altman plot, in which most of the big differences observed between CT and MR would occur when CT measurement is at one of the extremes of its range, since it is far less likely for MR measurement to be a nearby value; and this structure is not seen. Therefore, it is more likely that the increased variability is due to CT and MR surfaces being somewhat different due to the reconstructed CT and MR bone surfaces being somewhat different. A probable cause of this is geometric distortion by MR magnetic field inhomogeneity which would cause variability across participants and across imaging sites.

In contrast to the B-score, the tibia shape z-scores were somewhat lower from MR images, especially for the higher shape scores, with a bias of 0.424. Shape measures of the proximal tibia are somewhat more variable than those of the distal femur, with an SDD of 0.373, possibly because of the comparatively smaller surface area of this bone. Analysis of the tibia bone has been less well developed, and the reason for the bias here warrants further investigation.

CT and MR bone area measures also were all strongly correlated and all exhibited a negative bias indicating that the measures from MR images were consistently smaller than those from CT images. Because the two modalities measure different physical properties of materials, it is likely that the bone surface identified using MR and CT will be at slightly different positions within the bone/soft-tissue (cartilage) boundary. While the bone surface in an MR image will be indicated by a boundary between cortical bone with little water content, and cartilage which has a higher water content, the bone surface in a CT image is defined by the differing electron densities of cortical bone and cartilage. The consistent negative bias across all the bone area measures would indicate a MR bone boundary surface *inside* that of the CT boundary. This is also consistent with the larger 3DJSW measures (positive bias) from MR in comparison to CT.

In all measures, the analysis of the limited KLG 2&3 subset indicated a remarkably similar pattern and magnitude of the various statistical measures except for somewhat higher coefficient of determination and concordance correlation coefficient values in both the lateral and medial 3DJSW measurements.

Because MRI is not typically used to examine bony structures, there are few studies comparing the geometry of bone surfaces described by segmentation from CT or MR imaging. However, the use of imaging to generate 3D bone models by 3D printing for use in surgical planning and prostheses, including the design of patient-specific cutting guides and implants routinely uses CT, and the radiation dose from CT imaging can be a concern for elective surgery, prompting several studies comparing cadaveric bone model surfaces derived from CT and MR in the lower limb [36–39]. Although these studies all use different MR sequences, segmentation methodology, surface alignment algorithms, and surface comparison measures; MR and CT surfaces were found to be of comparable sub-millimeter accuracy with no significant differences in surface error. However, consistent with our results, the MR-derived surfaces were all found to be inside the CT-derived surfaces. In a direct comparison of MR and CT, Neubert et al [39] found that MR-based bone models were slightly smaller than CT-based models for all of 3 different MR sequences. A comparison of MR and CT to reference models generated by digitizing bone surfaces free of soft tissue with a mechanical contact scanner by Rathnayaka et al demonstrated that 75.8% of the surface area of MRI models underestimated the CT models [36]. Using optical scans of cleaned bones as ground truth, Broeck et al found that 3D bone models created from CT images are an overestimation of the actual bone, and MRI segmentation results in a 3D bone model that is on average an underestimation of the cleaned bone [37].

In contrast to orthopedic surgical planning, CT is not commonly employed in DMOAD trials due ionizing radiation and lack of soft tissue contrast. However, CT is more widely available than MRI and may have some advantages in clinical trials in which measurements from bone but not from bone marrow lesions or soft tissues are used as endpoints. CT images tend to be less expensive to acquire and standardization of CT image acquisition across sites in a multicenter trial should be more easily achieved than for MRI because of their lower complexity in setting acquisition protocols and lack of potential geometric distortion. CT does have an additional advantage in the determination of bone mineralization, and the measurement of bone mineral density (BMD) has been used to investigate OA-associated changes including subchondral bone trabecular remodeling [25, 40]. In addition, CT arthrography is considered the imaging reference standard for *in vivo* assessment of cartilage thickness [41], although this does require injection of intra-articular contrast material. Iterative reconstruction techniques are available which substantially reduces the ionizing radiation dose. Cone beam CT (CBCT) machines are becoming available that not only expose a patient to a much lower dose than conventional CT for a bilateral knee scan (~0.1 mSv or around 2 weeks of background radiation) [42], but also introduce the prospect of weight-bearing image acquisition and standardized 3DJSW measurement, which may provide more information about the disposition of the osteoarthritis knee than supine imaging [24].

The positive bias found here for both medial and lateral 3DJSW is consistent with the MR-derived surface being within the CT-derived surface, resulting in greater distance between MR femoral and tibial surfaces. However, there are substantially different biases for medial and lateral 3DJSW with the medial side being wider by around 0.6 mm. It is possible that this can be explained by differences in knee positioning during CT or MR acquisition. The APPROACH study CT acquisition protocol stipulated both knees to be as straight as possible with the feet on a wedge to produce around 15^o^ of internal rotation at the hip. In contrast, the MR acquisition protocol was to rotate the leg so that the toes and patella faced upward, elevate knee by putting cushions below it so that the center of the knee is place in the center of the coil and avoid scanning the knee in a fully extended or hyperextended position. Therefore, the leg was imaged fully extended during a CT scan and partially flexed during an MR scan. In a supine (open-chain) knee, during last 15-20^o^ of knee flexion, anterior tibial glide persists on the tibial medial condyle because its articular surface is longer than that on the lateral side. Prolonged anterior glide on the medial side produces external tibial rotation of around 15^o^, this is known as the “screw-home mechanism” [43]. This has the effect of repositioning the medial tibial region of the 3DJSW measurement (Fig. 1) more below a more anterior aspect of the medial femur, whereas the repositioning of the lateral tibial 3DJSW region will be less pronounced. This has consequences for the measurement of 3DJSW of the supine knee, and careful attention should be paid to the reproducible positioning of the knee. Further validation of non weight-bearing 3DJSW is therefore warranted.

The main limitation of this study was the lack of sufficient CT data to construct a new version of the OA-vector using shape measures derived from CT images. It would have been useful to compare this approach to the method that was used here, which was to fit the MRI AAM to the CT AAM search result surface and then project the rusting shape into the MRI OA-vector space. There was also no test-retest repositioning image data for CT available, which meant that we could not determine SDD of CT as a repeatability measure. In terms of the 3DJSW analysis, the difference in leg position during image acquisition meant that comparison of modalities is probably unreliable.

## Conclusions

In conclusion, the femoral bone shape (B-score) may be reliably measured using either MR or CT with a small bias that could probably be ignored for all practical purposes. Because it is a parameterised shape measure rather than a geometric measure, the B-score has the advantage over bone area measures of being both scale-independent and robust to changes in the position of the surface boundary due to the choice of imaging modality. The 3DJSW is potentially useful in the determination of meniscal or cartilage changes from CT or MR images, but is a geometric measure and is therefore dependent upon the relative positions of the femur and the tibia during image acquisition.

## Supplementary Information

**Additional file 1: Table S1.** Bland-Altman bias (MR minus CT), limits of agreement and linear regression statistics for the comparison of various MR and CT derived measures for the limited KLG dataset (KLG 2–3). **Fig. S1.** CT vs MRI bone area TrFMed.tAB for the full dataset (KLG 0–4). Top: Bland-Altman (MR minus CT) plot; Bottom: linear regression. **Fig. S2.** CT vs MRI bone area TrFLat.tAB for the full dataset (KLG 0–4). Top: Bland-Altman (MR minus CT) plot; Bottom: linear regression. **Fig. S3.** CT vs MRI tibia shape z-score for the full dataset (KLG 0–4). Top: Bland-Altman (MR minus CT) plot; Bottom: linear regression. **Fig. S4.** CT vs MRI bone area MT.tAB for the full dataset (KLG 0–4). Top: Bland-Altman (MR minus CT) plot; Bottom: linear regression. **Fig. S5.** CT vs MRI bone area LT.tAB for the full dataset (KLG 0–4). Top: Bland-Altman (MR minus CT) plot; Bottom: linear regression.

## Data Availability

The data that support the findings of this study are available from the IMI-APPROACH Consortium but restrictions apply to the availability of these data, which were used under license for the current study, and so are not publicly available. Data are however available from the authors upon reasonable request and with permission of the IMI-APPROACH Steering Committee.
