# Supplementary material for: Comparison of 3D quantitative osteoarthritis imaging biomarkers from paired CT and MR images: data from the IMI-APPROACH study
Source: BMC Musculoskelet Disord. 2023 Jan 30;24:76. doi: 10.1186/s12891-023-06187-2 (PMC9885640; doi:10.1186/s12891-023-06187-2)
Supplement: Supplementary file 1 — Additional file 1: Table S1. Bland-Altman bias (MR minus CT), limits of agreement and linear regression statistics for the comparison of various MR and CT derived measures for the limited KLG dataset (KLG 2–3). Fig. S1. CT vs MRI bone area TrFMed.tAB for the full dataset (KLG 0–4). Top: Bland-Altman (MR minus CT) plot; Bottom: linear regression. Fig. S2. CT vs MRI bone area TrFLat.tAB for the full dataset (KLG 0–4). Top: Bland-Altman (MR minus CT) plot; Bottom: linear regression. Fig. S3. CT vs MRI tibia shape z-score for the full dataset (KLG 0–4). Top: Bland-Altman (MR minus CT) plot; Bottom: linear regression. Fig. S4. CT vs MRI bone area MT.tAB for the full dataset (KLG 0–4). Top: Bland-Altman (MR minus CT) plot; Bottom: linear regression. Fig. S5. CT vs MRI bone area LT.tAB for the full dataset (KLG 0–4). Top: Bland-Altman (MR minus CT) plot; Bottom: linear regression. [file 12891_2023_6187_MOESM1_ESM.docx]

# **Supplementary Materials**

| Measure | Bland-Altman bias [95% CI] | Bland-Altman limits of agreement | | R^2^ | CCC |
| --- | --- | --- | --- | --- | --- |
|  |  | Lower [95% CI] | Upper [95% CI] |  |  |
| Femur shape B-score | 0.049  [0.024, 0.122] | -0.971  [-1.098, -0.845] | 1.069  [0.942, 1.196] | 0.918 | 0.958 |
| Tibia shape z-score | -0.479  [-0.557, -0.400] | -1.579  [-1.715, -1.442] | 0.621  [0.485, 0.758] | 0.884 | 0.896 |
| MF.tAB  (mm^2^) | -65.807  [-73.589, -58.026] | -174.356  [-187.834, -160.877] | 42.741  [29.263, 56.219] | 0.972 | 0.966 |
| LF.tAB  (mm^2^) | -36.879  [-42.808, -30.951] | -119.580  [-129.849, -109.312] | 45.822  [35.553, 56.090] | 0.970 | 0.973 |
| MT.tAB  (mm^2^) | -6.658  [-11.438, -1.879] | -73.330  [-81.609, -65.052] | 60.014  [51.735, 68.292] | 0.962 | 0.979 |
| LT.tAB  (mm^2^) | -25.600  [-29.596, -21.604] | -81.342  [-88.264, -74.421] | 30.142  [23.220, 37.063] | 0.960 | 0.959 |
| TrFMed.tAB  (mm^2^) | -29.376  [-32.242, -26.510] | -69.354  [-74.318, -64.390] | 10.602  [5.638, 15.566] | 0.949 | 0.920 |
| TrFLat.tAB  (mm^2^) | -42.390  [-47.524, -37.255] | -114.015  [-122.908, -105.121] | 29.235  [20.342, 38.129] | 0.948 | 0.940 |
| Medial 3DJSW  (mm) | 0.947  [0.851, 1.044] | -0.404  [-0.572, -0.236] | 2.299  [2.131, 2.467] | 0.597 | 0.537 |
| Lateral 3DJSW  (mm) | 0.282  [0.152, 0.413] | -1.538  [-1.764, -1.312] | 2.103  [1.877, 2.329] | 0.448 | 0.649 |

**Table S1: Bland-Altman bias (MR minus CT), limits of agreement and linear regression statistics for the comparison of various MR and CT derived measures for the limited KLG dataset (KLG 2-3).**

| 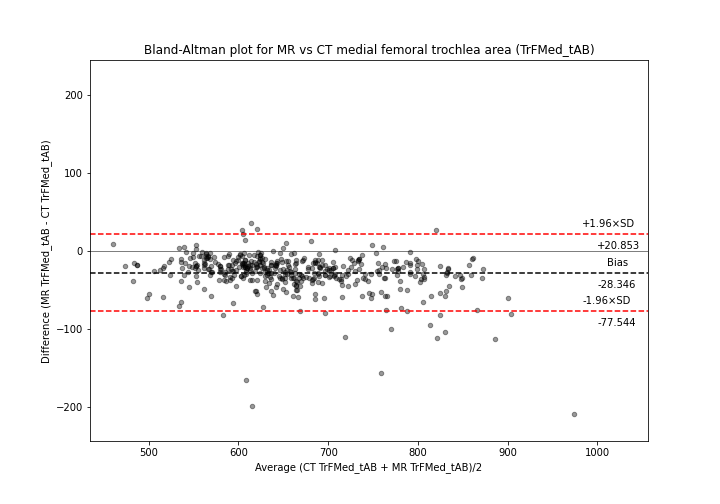 |
| --- |
| 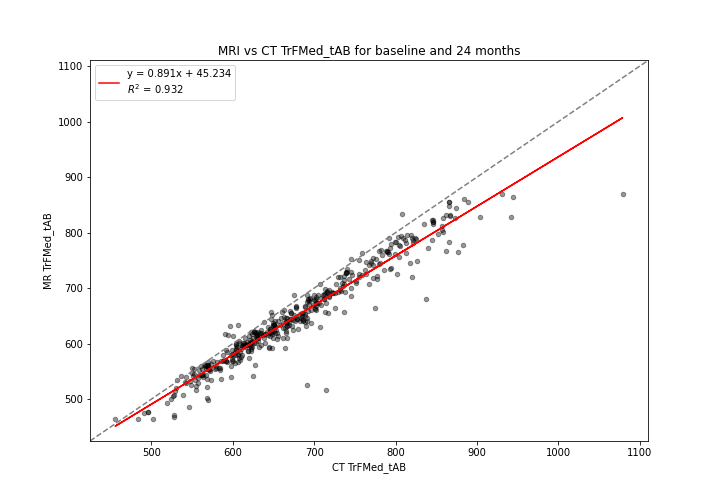 |

**Figure S1: CT vs MRI bone area TrFMed.tAB for the full dataset (KLG 0-4).** Top: Bland-Altman (MR minus CT) plot; Bottom: linear regression

| 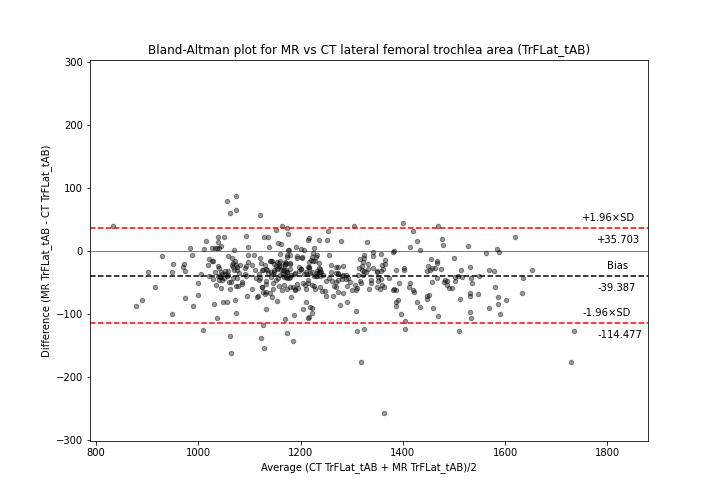 |
| --- |
| 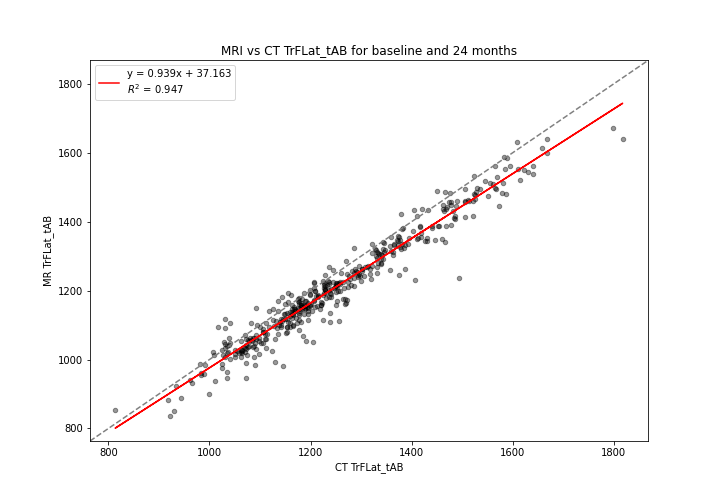 |

**Figure S2: CT vs MRI bone area TrFLat.tAB for the full dataset (KLG 0-4).** Top: Bland-Altman (MR minus CT) plot; Bottom: linear regression

| 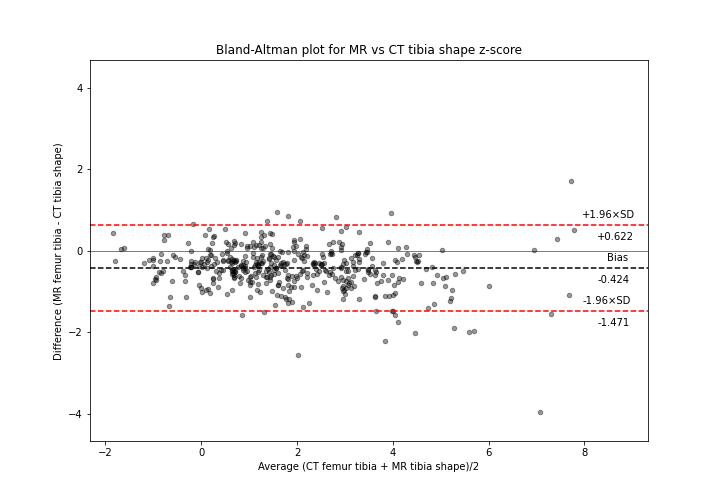 |
| --- |
| 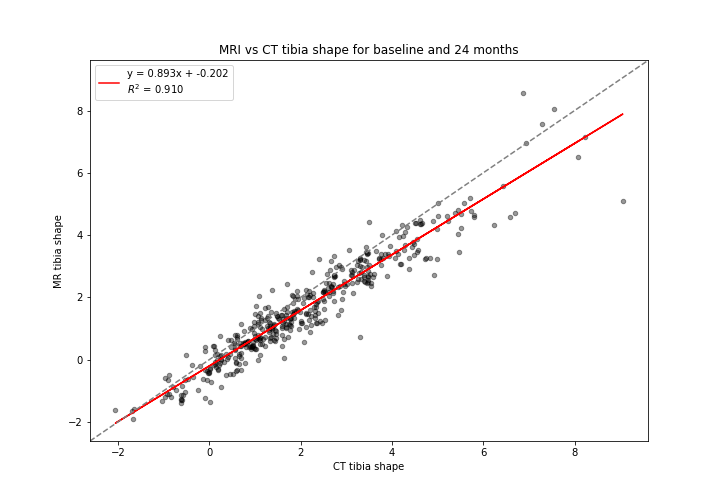 |

**Figure S3: CT vs MRI tibia shape z-score for the full dataset (KLG 0-4).** Top: Bland-Altman (MR minus CT) plot; Bottom: linear regression

| 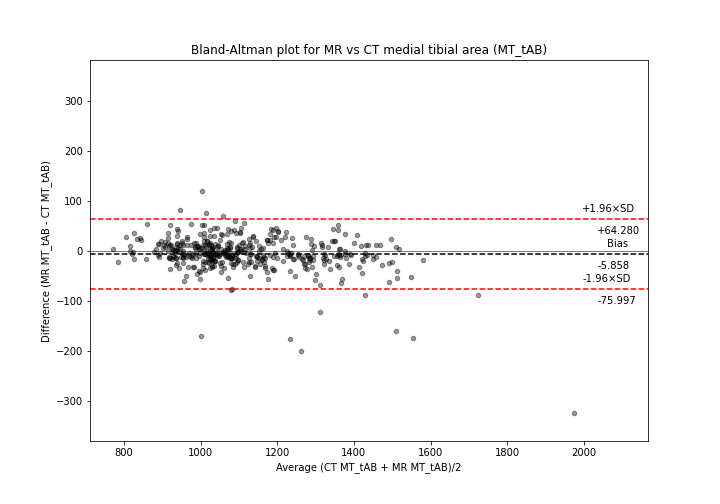 |
| --- |
| 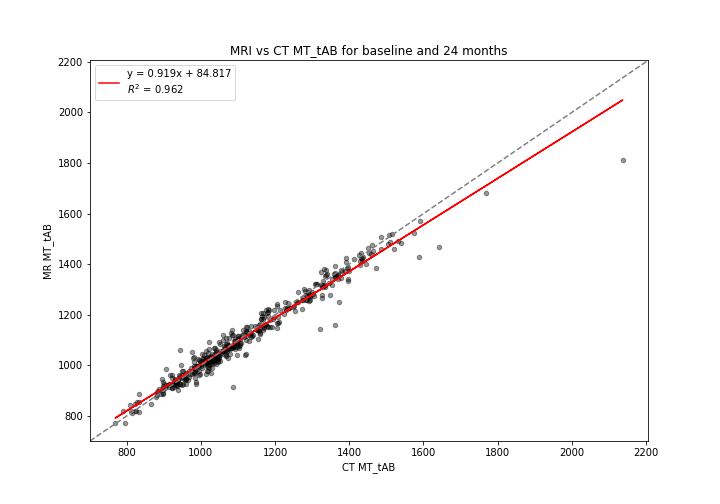 |

**Figure S4: CT vs MRI bone area MT.tAB for the full dataset (KLG 0-4).** Top: Bland-Altman (MR minus CT) plot; Bottom: linear regression

| 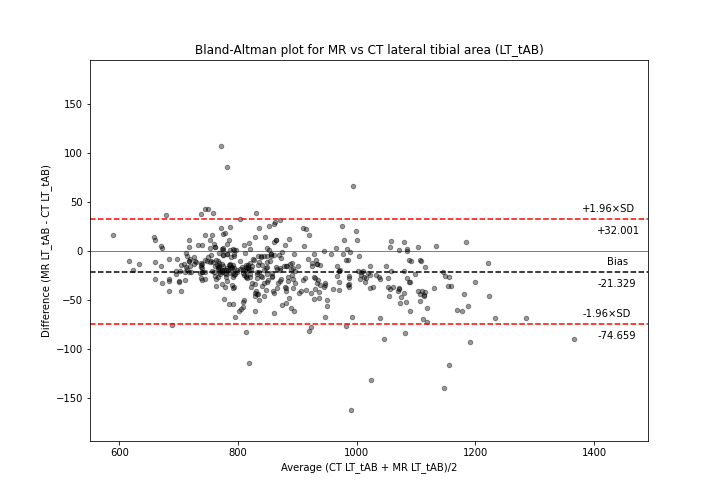 |
| --- |
| 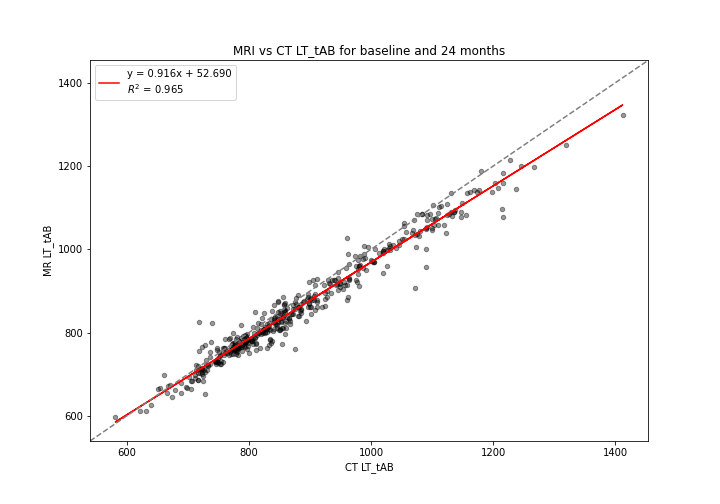 |

**Figure S5: CT vs MRI bone area LT.tAB for the full dataset (KLG 0-4).** Top: Bland-Altman (MR minus CT) plot; Bottom: linear regression
